# Relationships of sex hormones with muscle mass and muscle strength in male adolescents at different stages of puberty

**DOI:** 10.1371/journal.pone.0260521

**Published:** 2021-12-02

**Authors:** Yang Xu, Zhigang Wen, Kaili Deng, Ran Li, Qing Yu, Su-Mei Xiao

**Affiliations:** 1 Department of Epidemiology, School of Public Health, Sun Yat-sen University, Guangzhou, China; 2 Department of Endocrinology, Jiangmen Central Hospital, Affiliated Jiangmen Hospital of Sun Yat-sen University, Jiangmen, Guangdong, China; 3 Guangdong Provincial Key Laboratory of Food, Nutrition and Health, School of Public Health, Sun Yat-sen University, Guangzhou, China; University of Houston, UNITED STATES

## Abstract

This study analysed the associations of sex steroids with fat-free mass (FFM) and handgrip strength in 641 Chinese boys. Serum total testosterone (TT) and oestradiol were measured by chemiluminescence immunoassay. Free testosterone (FT) and oestradiol were calculated. FFM and handgrip strength were measured by bioelectrical impedance analysis and a hand dynamometer, respectively. Generalised additive models and multiple linear regression were used to explore the relationships. A subgroup analysis was conducted in early-mid pubertal and late-post pubertal groups. Age, height, weight, physical activity, intake of dietary protein and/or stage of puberty were adjusted. TT and FT were positively related to FFM and handgrip strength, with a curvilinear relationship being detected for handgrip strength (*p*<0.050). This curvilinear relationship was only observed in the late-post pubertal group, suggesting a potential threshold effect (FT>11.99ng/dL, β = 1.275, *p* = 0.039). In the early-mid pubertal group, TT and/or FT were linearly or near-linearly related to FFM or handgrip strength (β = 0.003–0.271, *p*<0.050). The association between FT and FFM was stronger than that in the late-post pubertal group. This study found that serum T had different associations with muscle parameters in Chinese early-mid pubertal and late-post pubertal boys. In the late-post pubertal boys, serum T was curvilinearly related to muscle strength with a threshold effect and its link with muscle mass was weaker.

## Introduction

Adequate muscle mass and muscle strength are essential to maintaining optimal health throughout life [1]. Studies have observed that muscle mass and muscle strength increase rapidly from childhood to puberty and reach a peak in early adult life, then decline with age from approximately the fifth decade [2–4]. Adolescents with low muscle strength are more likely to have low muscle strength in adulthood, a fact that suggests the need for concern about the levels of muscle power from a young age [5]. Moreover, in the child and adolescent populations, low muscle mass and muscle strength are found to be related to high risks of cardiovascular and metabolic diseases [1], and these may increase mortality and degrade the quality of life in the middle-aged and elderly population [6]. Inverse associations of muscle mass and muscle strength with metabolic risk factors, i.e., insulin resistance and blood lipids, have been demonstrated in adolescents by several studies [7]. Optimal development of muscle mass and muscle strength during adolescence is crucial not merely for general health, but also for preventing several common disorders, i.e., osteoporosis and sarcopenia, later in life [1]. In addition, optimal muscle growth is related to cognitive development and motor scores early in life [1, 8]. Therefore, maximising the gains in muscle mass and muscle strength in early life may be a critical strategy for reducing the risks of some common and increasingly prevalent disorders in later life.

In boys during puberty, sex hormones may have dramatic activating effects for promoting rapid accumulation of muscle mass and the acquisition of muscle strength [1, 2]. Testosterone (T) exerts important effects on muscles through various pathways. It can increase muscle mass by promoting myogenic differentiation of multipotent mesenchymal stem cells and by stimulating muscle protein synthesis [9]. Studies have reported that T is related to the accrual of muscle mass and the attainment of optimal muscle mass [10]. Puberty is associated with increased circulating T concentrations in adolescent boys [11]. Some studies in young men and adolescent boys have found that T is vital for the development and maintenance of muscle mass through its ability to stimulate whole-body protein synthesis and inhibit proteolysis, resulting in a net anabolic effect [12]. In addition, although the influence of oestradiol (E_**2**_) on muscle mass and muscle strength is less understood in men, the oestrogen receptor has been found to be expressed in male skeletal muscle [13]. Some studies have speculated that E_**2**_ could have an anabolic effect on muscle mass and could improve muscle strength in men and boys [13].

The influence of the serum concentrations of sex hormones on muscle mass and muscle strength in adolescent boys remains unclear due to the limited number of studies [14–16]. In adult men, the age-related decline in serum T concentrations has been implicated in reduced muscle mass and muscle strength [17]. T has also been used as a clinical supplement for older men to promote muscle mass gain [18]. Moreover, several studies in adult men have reported that the positive relationship between serum T and muscle strength could be nonlinear. In the Longitudinal Ageing Study Amsterdam, Schaap et al. found that only the concentration of total T (TT) in the 4th quartile group was related to muscle strength in 623 men [19]. The concentrations of sex hormones are among the factors that differ most prominently between the different stages of puberty. The results of these studies could imply that the effect of T on muscle varies between individuals at different stages of puberty. To date, no study has explored the relationship of serum T with muscle mass and muscle strength at different stages of puberty. As for serum E_**2**_, one study in 12.9-year-old boys and several studies in adult men observed that it was not associated with muscle parameters [14, 20]. In contrast, Vandenput et al. found that high serum E_**2**_ concentration was associated with higher lean mass in 3,014 Swedish adult men [21]. Understanding the impacts of sex hormones on muscles in adolescents may be indispensable for the optimal development of muscle mass and muscle strength.

Therefore, in this study, we first explored and investigated the relationships of serum T and E_**2**_ concentrations with muscle mass and muscle strength in 641 Chinese boys aged 11–18 years using generalised additive models as well as multiple linear regression, and further examined the associations between sex hormone concentrations and two muscle parameters at different stages of puberty.

## Materials and methods

### Study population

All participants were students at a secondary school in Jiangmen, China. The volunteers were recruited by posting advertisements and inviting them to attend a health talk presented by the investigators in each class at the school in 2015. The followed exclusion criteria were imposed: 1) a history of disorder or medication that may lead to abnormal sex steroid concentrations or muscle metabolism, i.e., musculoskeletal disorders, taking oestrogen, androgen or growth hormone treatment; 2) psychiatric or behavioural disorders; 3) critical illness, i.e., cancer; 4) contraindications for bioelectrical impedance analysis (BIA), i.e., implanted pacemaker, cardioverter defibrillator or diseases affecting the electrical resistance of the skin. However, no student had to be excluded due to these conditions. We assumed that the correlation coefficients between sex hormone and muscle mass, muscle strength were 0.38 and 0.45 in boys, respectively. The type I error rate was less than 0.05 (α = 0.05), and the power of test was 90% (β = 0.10). The maximum required sample size was 199. Although 697 participants were initially recruited, 56 of them were excluded due to missing data on their sex steroids. Finally, 641 participants were included in this analysis. This study was approved by the Ethics Committee of Sun Yat-sen University, and written informed consent was obtained from all participants and their parents or legal guardians.

### Measurement of muscle parameters

The total-body fat-free mass (FFM) of the subjects was measured using BIA (InBody230, Biospace, Seoul, Korea), with 10 impedance measurements at two frequencies (20 and 100 kHz). The subjects were asked to refrain from vigorous exercise for 30 minutes before the test. The *in vivo* coefficient of variation (CV) for the FFM was 0.3%. All tests were performed by the same investigator, and the operation followed the standard procedures. The handgrip strength was measured by a hand dynamometer (EH101, CAMRY, Guangdong, China). The handgrip strength of every individual was measured for each hand twice, alternating sides between each measurement. The maximum value of handgrip strength was recorded. Due to the difference in palm size among the individuals, the appropriate handle distance was adjusted specifically for each subject before each test to obtain accurate measurement values. The subjects were asked not to swing their arms, bend their arms or stoop during the test. The precision of handgrip strength was 2.6%.

### Hormone assays

Approximately 3-mL venous blood samples were collected from all subjects under fasting conditions (12–14-hour fasting) and stored at −80°C. The concentrations of serum T, E_**2**_ and sex hormone-binding globulin (SHBG) were measured by a fully automated chemiluminescent immunoassay system (Centaur XP, Siemens, Erlang, Germany). The albumin concentration was measured by the bromocresol green assay using a commercial kit (FUJIFILM Wako Pure Chemical Corporation, Osaka, Japan). All samples were analysed in the same laboratory in accordance with the standard procedures. The *in vivo* CV values for all measurements were within 3.0%. The free T (FT) and free E_**2**_ (FE_**2**_) concentrations were calculated using a previously validated equation established by Vermeulen et al. [22] taking account of the concentrations of TT, total E_**2**_ (TE_**2**_), SHBG and albumin.

### Assessment of covariates

A face-to-face interview was conducted at the school by trained personnel using a structured questionnaire to collect information of the subjects’ demographic characteristics, lifestyle habits, history of disease and medications. Height and weight were measured using standardised equipment. Height was measured to 0.1-centimetre (cm) accuracy without shoes. Weight was measured to the nearest 0.1 kg without shoes or any heavy clothing. Body mass index (BMI) was calculated by dividing weight (kg) by height squared (m^**2**^). Physical activity was assessed using the modified Chinese version of the Children’s Leisure Activities Study Survey questionnaire [23] and expressed as metabolic equivalent (MET·h/d). Intake of dietary protein was estimated using a 3-day 24-hour dietary recall record and the Chinese Food Composition Table. Pubertal development stage was assessed using a Chinese version of the self-reported Pubertal Development Scale (PDS) [24]. Pubertal data were dichotomised as early-mid pubertal vs late-post pubertal according to the PDS data and the reference intervals of TT levels at different pubertal stages in boys [25, 26]. The concentrations of fasting plasma glucose and insulin were measured by spectrophotometer (Nanodrop2000, ThermoFisher Scientific, Massachusetts, USA) and fully automated chemiluminescent immunoassay system (Centaur XP, Siemens, Erlang, Germany), respectively. Insulin resistance index was calculated by homeostasis model assessment of insulin resistance (HOMA-IR) as (fasting insulin mU/L) × (fasting glucose mmol/L) / 22.5.

### Statistical analysis

Continuous variables were presented as mean and standard deviation (SD), or median and interquartile range. To investigate the association of sex steroids with muscle mass and muscle strength, the generalised additive model (GAM) was first adopted to explore the functional form of the association between the sex steroids and the two muscle parameters in all subjects. The GAM analysis was also conducted in subgroups classified according to different stages of puberty. Furthermore, the specific estimation and inference of the associations between the sex steroids and the muscle parameters were detected with a multiple linear regression analysis. If nonlinearity was detected in the GAM analysis, a two-piecewise multiple linear regression analysis was performed to determine the break-point of the association between the sex hormone and the muscle parameter [27]. Confounding factors were selected based on the previous literatures and the results of univariate analysis. The confounding factors, i.e., age, height, weight, physical activity, intake of dietary protein and stage of puberty (not for the subgroup analysis), were adjusted in all of the models. Besides the stage of puberty, all other confounding factors were included in the model as continuous variables. All statistical analyses were conducted with R software (version 3.5.1, Vienna, Austria) and SPSS (v20.0, Chicago, Illinois, USA). A two-sided *p*-value of less than 0.05 was considered as statistically significant.

## Results

### Basic characteristics of the studied population

The basic information of the subjects is shown in Table 1. The age of the participants ranged from 11 to 18 years, and the median age was 15.6 years. The mean (SD) values of height and weight were 165.26 (10.57) cm and 52.27 (12.31) kg, respectively. The average BMI was 18.95 kg/m^**2**^. The interquartile range of the total physical activity was 14.45 to 23.76 MET·h/d, with the median value being 18.46 MET·h/d. The mean value of intake of dietary protein was 90.60 g/d. The median values of TT, TE_**2**_ and SHBG were 350.00 ng/dL, 39.27 pg/mL and 32.50 nmol/L, respectively. The mean (SD) values of the FFM and handgrip strength were 44.83 (9.06) kg and 33.08 (9.80) kg, respectively.

**Table 1:** Basic characteristics of the studied sample.

Variable	Mean ± SD/Median (25th–75th)		
	Total	Early-mid pubertal	Late-post pubertal
	(n = 641)	(n = 224)	(n = 417)
Age (years)	15.6 (13.6, 16.1)	13.0 (12.8, 13.8)	15.9 (15.6, 16.2)
Height (cm)	165.26±10.57	155.12±9.62	170.69±6.12
Weight (kg)	52.27±12.31	43.22±10.61	57.11±10.27
BMI (kg/m^2^)	18.95±3.24	16.92±3.37	19.55±3.01
Total physical activity (MET·h/d)	18.46 (14.45, 23.76)	18.45 (14.51, 23.53)	18.46 (14.44, 23.80)
Dietary protein intake (g/d)	90.60±27.88	88.72±34.21	97.38±30.92
HOMA-IR	1.67 (1.45, 1.89)	1.44 (1.26, 1.74)	1.73 (1.55, 1.93)
TT (ng/dL)	350.00 (196.00, 460.50)	161.00 (50.00, 283.75)	404.00 (313.00, 505.00)
TE_2_ (pg/mL)	39.27 (28.18, 50.13)	26.91 (19.18, 44.26)	42.56 (35.22, 50.91)
FT (ng/dL)	6.71 (3.06, 9.55)	2.52 (0.66, 4.91)	8.52 (6.28, 10.59)
FE_2_ (pg/mL)	0.70 (0.48, 0.93)	0.43 (0.28, 0.75)	0.79 (0.63, 0.99)
SHBG (nmol/L)	32.50 (24.70, 45.89)	45.77 (29.48, 65.27)	30.32 (22.98, 38.68)
FFM (kg)	44.83±9.06	36.37±7.35	49.54±6.37
Handgrip strength (kg)	33.08±9.80	24.07±6.54	38.03±7.28

Note: Data were presented as mean ± standard deviation (SD), or median and interquartile range. BMI, body mass index; TT, total testosterone; TE_**2**_, total oestradiol; FT, free testosterone; FE_**2**_, free oestradiol; SHBG, sex hormone-binding globulin; MET, metabolic equivalent; FFM, fat-free mass; HOMA-IR, homeostatic model assessment-insulin resistance.

### GAM analysis of sex steroids with muscle parameters

**Fig 1:**
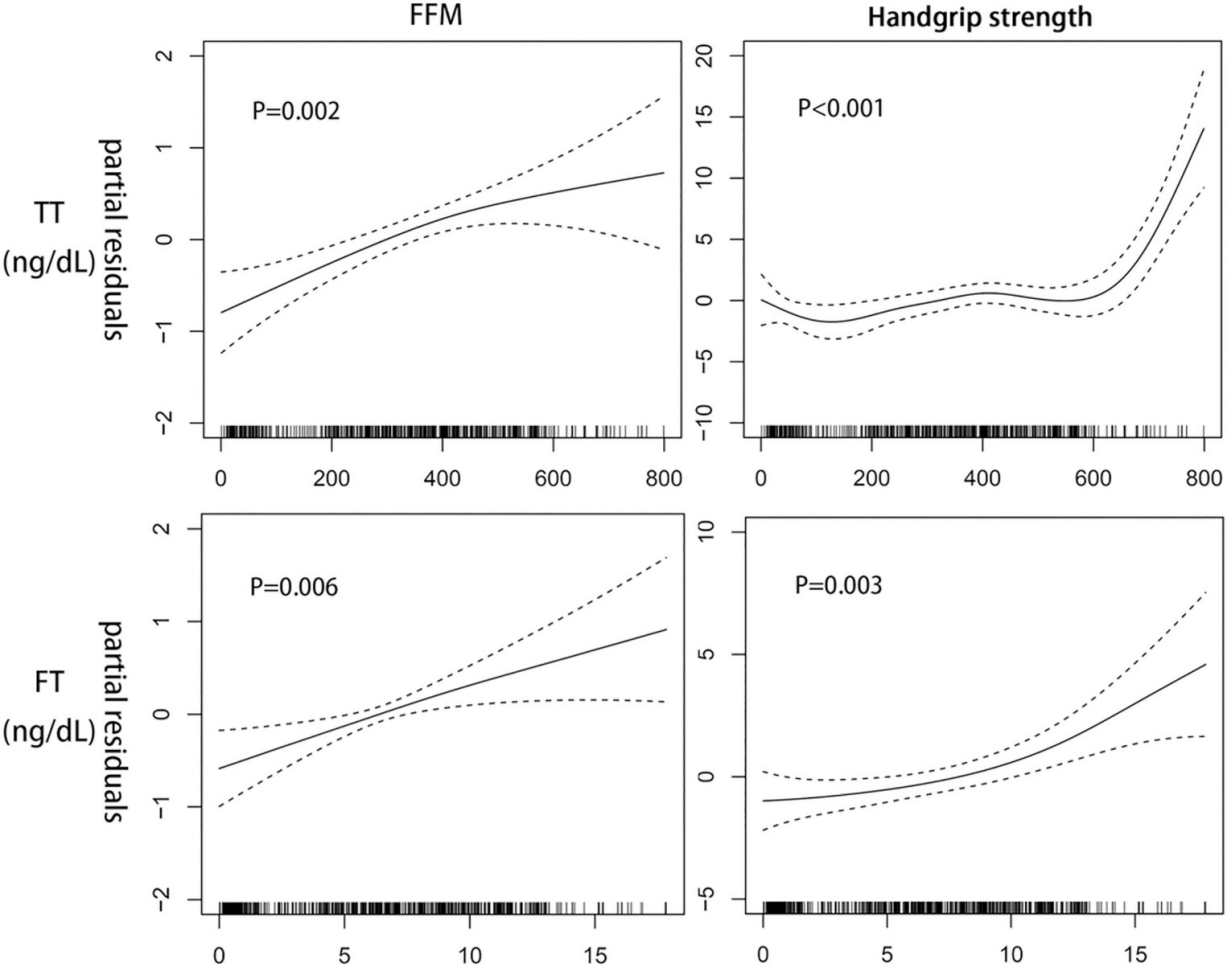
Associations between TT, FT and fat-free mass (FFM), handgrip strength estimated using the generalised additive regression models (n = 641). Dotted lines represent the 95% confidence intervals. The adjusted covariates included age, height, weight, physical activity, intake of dietary protein and stage of puberty. TT: total testosterone; FT: free testosterone.

Fig 1 depicts the results of the GAM analysis in all subjects (n = 641), with adjustment for age, height, weight, physical activity, intake of dietary protein and stage of puberty. The FFM was linearly or near-linearly correlated with the concentrations of TT (*p* = 0.002) and FT (*p* = 0.006), respectively. The handgrip strength had a curvilinear relationship with the concentrations of TT and FT (*p* < 0.050), in which the curves became steeper when the TT and FT concentrations were more than approximately 600 ng/dL and 10 ng/dL, respectively. In this study, HOMA-IR showed a marginal significant inverse correlation with muscle strength (*r* = -0.06, *p* = 0.082) and TT (*r* = -0.04, *p* = 0.160). HOMA-IR was further added as a covariate in the statistical model, and those results of the associations of TT and FT with muscle parameter remained similar. No significant relationship was observed for the concentrations of TE_**2**_ or FE_**2**_ with respect to either of the muscle parameters in the GAM analysis (*p* > 0.050).

**Fig 2:**
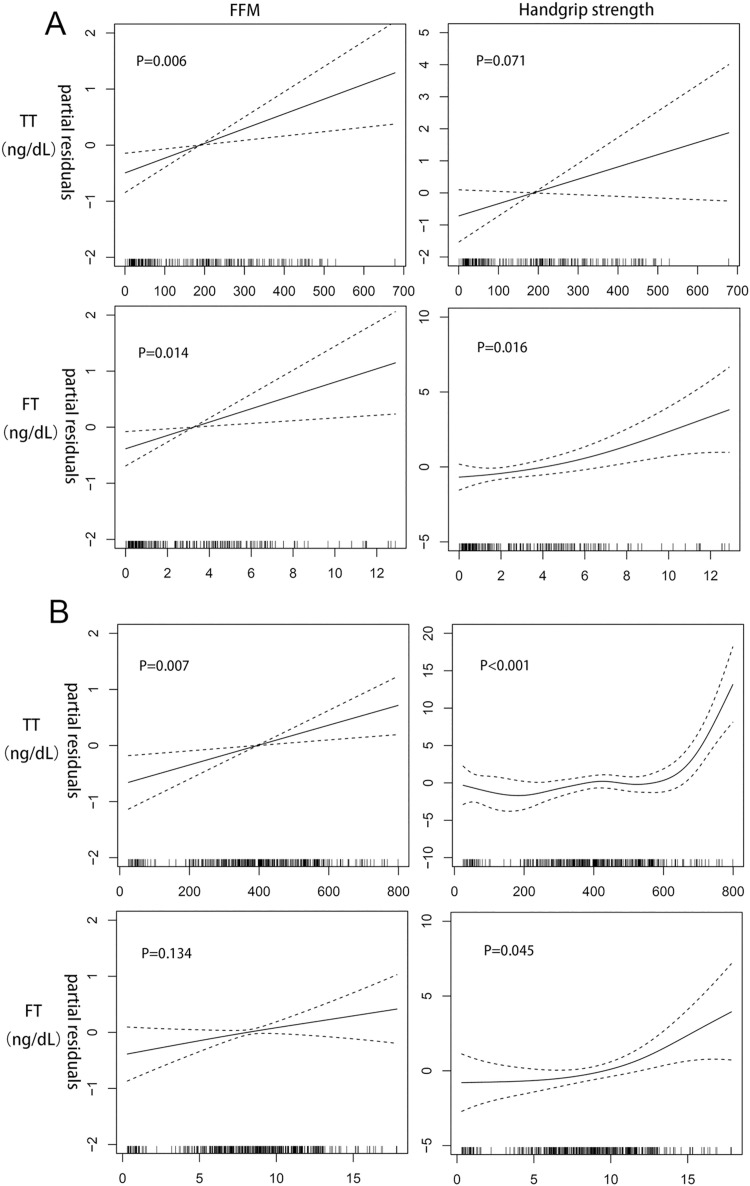
The results of subgroup analyses using the generalised additive regression models. A. Associations between TT, FT and fat-free mass (FFM) in the early-mid pubertal group. B. Associations between TT, FT and FFM, handgrip strength in the late-post pubertal group. Dotted lines represent the 95% confidence intervals. Covariates included age, height, weight and physical activity and intake of dietary protein. TT: total testosterone; FT: free testosterone.

Subgroup analyses for the association of sex steroids with the FFM and handgrip strength were subsequently performed in the early-mid pubertal group (n = 224) and the late-post pubertal group (n = 417). As shown in Fig 2, the results of the GAM in the early-mid pubertal group showed linear or near-linear relationships between the FT concentration and FFM (*p* = 0.014), the FT concentration and handgrip strength (*p* = 0.016), and the TT concentration and FFM (*p* = 0.006). In the late-post pubertal group, the FFM exhibited a significant linear relationship only with the TT concentration (*p* = 0.007). Although a linear trend between the FT concentration and FFM was observed, it was not statistically significant (*p* = 0.134). Handgrip strength showed curvilinear relationships with the concentrations of TT (*p* < 0.001) and FT (*p* = 0.045) in the late-post pubertal group. However, the TE_**2**_ and FE_**2**_ concentrations were not related to the FFM or handgrip strength in the late-post pubertal group (*p* > 0.050).

### Linear regression analysis of sex steroids with muscle parameters

Linear regression analysis was conducted to make the specific estimation and inference of the associations of the TT and FT concentrations with both muscle parameters in the early-mid pubertal group, as well as with the FFM in the late-post pubertal group (Table 2). For the early-mid pubertal group, the TT and FT concentrations were positively related to the FFM (β_**TT**_ = 0.003, β_**FT**_ = 0.131, *p* < 0.050). Handgrip strength also showed a positive association with the FT concentration (β_**FT**_ = 0.271, *p* = 0.016). No significant association was observed between the TT concentration and handgrip strength (*p* > 0.050). For the late-post pubertal group, a positive association of the FFM was observed with the TT concentration (β = 0.002, *p* = 0.023), but not with the FT concentration (*p* > 0.050). These results of linear regression were consistent with those of the GAMs.

**Table 2:** Results of associations between TT, FT and FFM, grip strength in the multiple linear regression analyses.

	β	SE	P
**Early-mid pubertal group (n = 224)**			
*FFM*			
TT (ng/dL)	0.003	0.001	**0.019**
FT (ng/dL)	0.131	0.055	**0.018**
*Handgrip strength*			
TT (ng/dL)	0.004	0.002	0.101
FT (ng/dL)	0.271	0.112	**0.016**
**Late-post pubertal group (n = 417)**			
*FFM*			
TT (ng/dL)	0.002	0.001	**0.023**
FT (ng/dL)	0.049	0.034	0.146

Note: The confounding effects of age, height, weight and physical activity and intake of dietary protein were adjusted in the analysis. TT, total testosterone; FT, free testosterone; FFM, fat-free mass; β, regression coefficient; SE, standard error; bold, p<0.05.

### Threshold analysis for serum T and handgrip strength in the late-post pubertal group

The relationship between the FT concentration and handgrip strength was found to be curvilinear in the late-post pubertal group according to the GAMs. Therefore, a two-piecewise multiple linear regression analysis was conducted to investigate the potential threshold effects of the FT concentration on handgrip strength. The results showed that the break-point was at 11.99 ng/dL. As shown in Table 3, the handgrip strength showed a significant positive association with the FT concentration in the individuals with FT concentrations above 11.99 ng/dL (β = 1.275, *p* = 0.039), after adjusting for the confounding factors. Handgrip strength had no association with the FT concentrations in the subjects with FT concentrations below that value (*p* > 0.050). The TT concentration corresponding to a FT concentration of 11.99 ng/dL in this population was approximately 708.00 ng/dL based on the serum SHBG and albumin concentrations. An analogous exploratory analysis performed for the TT concentration with a split point of 708.00 ng/dL showed similar trends.

**Table 3:** Threshold effect analysis of FT on handgrip strength using two-piecewise multiple linear regression models in the late-post pubertal boys (n = 417).

FT (ng/dL)	β	SE	P
< 11.99	0.079	0.116	0.500
>11.99	1.275	0.624	**0.039**

Note: The confounding effects of age, height, weight, physical activity and intake of dietary protein were adjusted in the analysis. FT, free testosterone; β, regression coefficient; SE, standard error; bold, p<0.05.

## Discussion

In this study, we investigated the associations of sex steroids with muscle parameters in 641 Chinese male adolescents. The results indicated that the concentrations of TT and FT were positively related to the FFM and handgrip strength, and a curvilinear relationship of the serum T concentration with handgrip strength was detected. Further subgroup analyses found that this curvilinear relationship was only observed in the late-post pubertal group, suggesting a potential threshold effect. In addition, compared with the early-mid pubertal group, the strength of association for the serum T concentration and the FFM became weaker in the late-post pubertal group.

This study supported the hypothesis that a high concentration of serum T contributes to the gain of muscle mass and muscle strength in male adolescents, consistent with some previous studies [14, 28]. A study of the ‘Children of 1997’ birth cohort in Hong Kong found that the FT concentration had a positive association with skeletal muscle mass in adolescent boys [14]. Hou et al. found that a higher TT concentration was related to a larger value of the skeletal muscle index in 278 boys aged 15 years [28]. Ramos et al. reported that the TT concentration was positively correlated with the strength of knee extensors in 11–18-year-old boys [16]. Evidence indicates that the acquisition of muscle mass and muscle strength takes place largely during puberty and is promoted by sex steroids [29, 30]. Both animal and human studies have demonstrated that the androgen receptors (ARs) are highly expressed in muscles and that the androgen–AR pathway is essential for increases in muscle strength and muscle mass [31]. Dramatic hormonal fluctuations in puberty are accompanied by marked changes in body composition, e.g. lean mass [29]. During puberty, TT production increases from approximately 0.3 mg/d to 7 mg/d with an increase in circulating T concentrations [32]. This may stimulate the increases in the numbers and sizes of muscle fibres and the numbers of muscle satellite cells and myonuclei [9]. Evidence also demonstrates that T can promote mitochondrial biogenesis and myoglobin expression [9], which might improve the muscle strength.

The results of the present subgroup analysis suggested that T contributed to the muscle strength but not the muscle mass in the late-post pubertal boys. In the early-mid pubertal group, however, the concentration of FT, as the most biologically active fraction, was positively associated with both the FFM and handgrip strength. These findings might imply that the links between T and muscle mass might become weaker in the late-post pubertal boys. A study including 777 Chinese male subjects aged 5–19 years observed a sharp increase in lean mass until the age of 14 years, after which the slope of the increase in lean mass became less steep and flattened out at the age of 16–19 years [33]. Some other studies have also reported similar findings. Veldhuis et al. found that the FFM was stable by 17–19 years in Caucasian boys [2]. For muscle strength, however, the findings of a cohort study indicated that it continuously increased in young men between 19 and 21 years of age [34]. Other studies have also found that the handgrip strength may increase continuously to a peak in early adult life around 30 years, and remain at the peak value through to midlife [3]. These findings suggest that the increases in muscle mass and muscle strength do not occur in parallel in the late-post pubertal boys. Furthermore, a study of the MrOS Hong Kong cohort comprising 1,489 adult men found that the effect of T on muscle strength might be independent of muscle mass [17]. Some studies have reported that other factors, e.g. neuromuscular adaptations, besides the increase in muscle mass could account for part of the increase in muscle strength during adolescence [35]. Therefore, the contribution of T to the increase in muscle strength might be less closely related to muscle mass in the late-post pubertal boys.

Our data also revealed that the relationship between the serum T concentration and muscle strength was curvilinear in the late-post pubertal group. To the best of our knowledge, no previous study has reported a nonlinear relationship between serum T and muscle parameters in adolescents. In this study, only for the late-post pubertal boys in the 87th percentile of FT concentration (above 11.99 ng/dL), their FT concentration had an association with the handgrip strength. This FT concentration corresponds to a total T concentration of approximately 708.00 ng/dL. This finding might imply that only those very few late-post pubertal adolescent boys with very high T concentrations could reap the benefits of T on muscle strength. Exercise is the general adopted method for substantial gains in muscle strength. Studies have found that men with high concentrations of serum T and high rates of exercise had the greatest increase in muscle strength [36]. Moreover, other studies have found that after exercise, the expression of ARs can be upregulated by muscle contraction, which might enhance T uptake to the muscle and potentially exert anabolic effects [37]. These findings could support the interactive effect of T and exercise on muscle strength. In this study, high serum T concentrations had a linear relationship with muscle strength in the early-mid pubertal boys, but among the late-post pubertal boys, serum T only contributed to muscle strength for those very few boys in the 87th percentile of FT concentration. These results might suggest that exercise intervention in the early-mid pubertal boys might have greater effects on muscle strength than in the late-post pubertal boys for most individuals.

This study investigated the relationships of the serum T concentration with muscle parameters in a larger sample size compared with previous studies, and revealed the different contributions of serum T to muscle mass and muscle strength between early-mid pubertal and late-post pubertal boys for the first time. This study also has some limitations. First, the FFM was measured using BIA, rather than magnetic resonance imaging (MRI), which is a gold standard method for measuring muscle mass. MRI has high accuracy and ability to differentiate between tissue types. However, the apparatus is not portable and requires highly specialized personnel, and the cost is high. These limit its use in a large-scale epidemiological study in normal adolescents [38]. BIA has been validated by MRI in this aspect, and the results indicated it as a good choice for a portable method of muscle mass measurement for Chinese. The concordance coefficient between BIA and MRI for the FFM is satisfactory (0.85) [39]. Second, although mass spectrometry is often taken as the gold standard method to measure sex steroid concentrations, this study used automatic immune analysers. Nonetheless, these are sufficiently reliable for epidemiological studies in general populations and are also widely used in clinical and reference laboratories [40]. Third, the use of handgrip strength test only cannot give a comprehensive assessment of muscle strength. For example, the hand grip strength may not be a good measure for the strength of lower extremity. Nonetheless, the hand grip strength is a reliable and simple surrogate of upper extremity strength, and some studies found that it correlates well with other muscle strength tests such as the knee extension strength [41, 42]. Fourth, the clinical assessment with Tanner staging is widely recognized as the gold standard method for assessing pubertal development [43]. However, the privacy concerns make it difficult to obtain consent from the adolescents and their parents. PDS may be a useful and reliable alternative method for assessing puberty in adolescents, which is a low-burden self-reporting method without the physical examination [44]. Evidences supported that PDS had strong internal consistency and reliability (Cronbach’s alpha: 0.83), and substantial correlation (0.82) with Tanner staging by clinical assessment in boys [45]. Discrepancies could exist between the self-reported and the actual pubertal stage in this study. Nonetheless, in order to increase the accuracy, besides the self-reported PDS data, the information of the serum testosterone levels was also adopted for the classification of pubertal stages according to the reported reference intervals at different pubertal stages. Finally, the changes in the FFM and handgrip strength over time were not available in this cross-sectional study. Further longitudinal studies are warranted for investigating the relationships of sex steroids with the changes in muscle mass and muscle strength in adolescent boys.

In conclusion, this study indicated that the serum T concentration as a positive predictor had different associations with muscle mass and muscle strength depending on the pubertal development stage of Chinese male adolescents. In the late-post pubertal boys, the links between serum T concentration and muscle mass became weaker compared with that in the early-mid pubertal boys, and a curvilinear relationship between the T concentration and muscle strength was found with a threshold effect. These findings are expected to improve our understanding of the effects of sex steroids on muscle mass and muscle strength accrual during male adolescence and to provide useful information for the establishment of a prediction model for the peak value of muscle mass and muscle strength.
